# Relapsing polychondritis: focus on cardiac involvement

**DOI:** 10.3389/fimmu.2023.1218475

**Published:** 2023-09-12

**Authors:** Ruxue Yin, Mengzhu Zhao, Dong Xu, Qian Wang, Mengtao Li, Wen Zhang, Fengchun Zhang, Xiaofeng Zeng, Yuping Huo, Yong Hou

**Affiliations:** ^1^ Key Laboratory of Rheumatology and Clinical Immunology, Department of Rheumatology, Peking Union Medical College Hospital, Peking Union Medical College and Chinese Academy of Medical Science, Ministry of Education, National Clinical Research Center for Dermatologic and Immunologic Diseases, Ministry of Science & Technology, Beijing, China; ^2^ Department of Rheumatology, Jin Cheng People’s Hospital, Jincheng, Shanxi, China

**Keywords:** relapsing polychondritis, risk factor, prognosis, cardiac, neutrophil-to-lymphocyte ratio, central nervous system

## Abstract

**Background:**

Relapsing polychondritis (RP) with cardiac involvement may present with acute cardiovascular events, and may be associated with a negative prognosis. Herein, we analyzed the clinical characteristics of RP patients with cardiac involvement.

**Method:**

RP patients, hospitalized from December 2005 to December 2021 at Peking Union Medical College Hospital (PUMCH), were screened. Univariate and multivariate logistic regression analyses were used to statistically analyze the clinical characteristics of these patients.

**Results:**

The incidence of cardiac involvement in inpatients with RP was 24.1%. Univariate logistic regression analysis revealed age, central nervous system (CNS) involvement, neutrophil-to-lymphocyte ratio (NLR) > 6.41, and disease duration > 4 years as risk factors for cardiac involvement in RP. Conversely, the incidence of tracheobronchial and chest wall involvement was significantly lower in the group with cardiac involvement. Multivariate logistic regression confirmed that age, CNS involvement, NLR > 6.41, and disease duration > 4 years were independent factors for cardiac involvement. Subsequently, we identified five well-defined clinical patterns of RP, based on the involvement of different organs in our patients, and found that the heart-brain model was significantly mutually exclusive with the airway model.

**Conclusion:**

Occurrence of cardiac involvement in RP is associated with age, CNS involvement, NLR, and disease duration. It is mutually exclusive with airway-related involvement. Regular echocardiography and electrocardiography are necessary for patients with RP.

## 1 Introduction

Relapsing polychondritis (RP) is a rare inflammatory disease involving multiple organs, including the eyes, ears, nose, larynx, tracheobronchial tree, joints, kidneys, cardiovascular system, and nervous system. The pathogenesis of RP is currently unclear, although previous studies have indicated that both humoral and cell-mediated immune systems may be involved in its progression and development, and that cartilage-specific autoimmunity may play a critical role in the overall pathogenesis of RP (1, 2). Although RP can be present at any age, it develops most frequently between the ages of 40 and 60 years, with a male-to-female ratio of almost 1:1 (3). RP has an insidious and variable clinical presentation. In a previous study, French researchers identified three separate clusters in terms of clinical manifestations and prognosis, namely, hematologic, respiratory, and mild phenotypes, and suggested that cardiac involvement is a significant sign of poor prognosis in RP patients (4).

In clinical practice, it is commonly observed that cardiac involvement is generally insidious; however, cases of acute cardiovascular events have also been reported (5). We believe that the importance of cardiac involvement in RP may be underestimated and, in turn, has not received sufficient attention. To our knowledge, only one study has yet compared the differences in clinical presentation between patients with and without cardiac involvement, while studies comparing laboratory indices and detailed prognostic features are lacking. As such, current knowledge regarding RP with cardiac involvement remains insufficient.

The objective of this study was, therefore, to comprehensively clarify the risk factors of cardiac involvement. This is the most detailed large-scale study of RP cardiac involvement performed to date.

## 2 Methods

### 2.1 Patients

We searched the medical records of the Peking Union Medical College Hospital (PUMCH) for ICD codes indicating RP from December 2005 to December 2021, identifying 249 RP inpatients who were selected for preliminary analysis. All patients fulfilled the diagnostic criteria of RP described by the McAdam et al. (6) or those introduced by Damiani and Levine (7). Combinations of malignant tumors and other autoimmune diseases were excluded. In addition, RP patients who have concomitant cardiovascular diseases, which unequivocally lead to structural alterations in the cardiac, were excluded from the scope of this study. Finally, a total of 187 patients were included in the initial prognostic analysis. The clinical features of all patients, including age, sex, disease duration, organ involvement, risk factors for common cardiovascular diseases, and laboratory tests [including white blood cell (WBC), hemoglobin (Hb), platelet count (PLT), albumin (Alb), alanine transaminase (ALT), lactate dehydrogenase (LDH), C-reactive protein (CRP) level, and erythrocyte sedimentation rate (ESR)] were determined. In addition, novel inflammatory markers, including the platelet-to-lymphocyte ratio (PLR), neutrophil-to-lymphocyte ratio (NLR), and C-reactive protein to albumin ratio (CAR), have been shown to be associated with a variety of immune and heart diseases; therefore, we also included patients with these in our study. This study complied with the tenets set out by the Declaration of Helsinki, and all procedures were approved by the Ethics Committee of Peking Union Medical College Hospital. Written informed consent was obtained from all patients, and all data were analyzed anonymously.

### 2.2 Outcome assessment

Cardiac involvement included valve incompetence or regurgitation, aortic aneurysm, aortic root dilatation, pericarditis, myocarditis, right bundle branch, or atrioventricular block, except for other causes of pericarditis, myocarditis, or arteritis (5). The presence of cardiac involvement was assessed via transthoracic echocardiography and electrocardiography. Echocardiogram and ECG were interpreted independently by both an echographer, and a cardiologist. Standard definitions were applied to define the features associated with the disease. All organ involvement was determined by clinical presentation and ancillary investigations combined with assessment by the relevant specialists. To determine the prognostic factors of cardiac involvement, we divided RP inpatients into two groups according to the presence or absence of cardiac involvement. Clinical variables were subsequently compared between the two groups.

All patients were followed up for 1–3 years. Remission was defined in patients who became asymptomatic following a treatment regimen comprising a daily dose of less than 10 mg of corticosteroids for more than three months. Relapse was defined as severe impairment requiring a change in treatment or an increase in the daily dose of glucocorticoids to >0.5 mg/kg (8). Patients who experienced relapse or death were classified as having a poor prognosis.

### 2.3 Statistical analysis

All statistical analyses were performed using R software (version 4.20) and SPSS (version 21.0). For continuous variables, the receiver operating characteristic (ROC) curve was applied to determine the best cut off value. Univariate logistic analysis was applied to identify factors that could differentiate between patients with cardiac involvement and those without cardiac involvement; factors with p < 0.05 were incorporated into multivariate binary logistic regression to further identify independent factors. To better identify risk factors of cardiac involvement, we treated all continuous variables except age as dichotomous variables for the supplementary analysis, as follows: For parameters with specified normal ranges, such as WBC, PLT, Hb, NEUT, LDH, ALT, Alb, Ferrin, Cr, CK, CRP and ESR, etc., we cut-off at the upper or lower limit of the normal range. As there is no accepted cut-off point for CAR, PLR, NLR, and disease duration, the ROC curve was used to determine the most optimal segmentation point.

## 3 Results

### 3.1 Patient demographics and clinical manifestations

Of the 187 hospitalized RP patients, 104 were men and 83 were women. The mean age of the enrolled patients was 47 years. The incidence of cardiac involvement in inpatients with RP was 24.1% (45/187). Among the 45 RP patients with cardiac involvement, the male: female ratio was nearly 3:2, and the mean age was 51 years (range 10 to 74 years). The mean disease duration of RP was 2.17 years (range 2 months to 20 years).

Comparing patients with and without cardiac involvement, ocular involvement was seen in 52.2% vs. 35.9% of patients, nasal involvement in 52.2% vs. 57.7%, saddle nose involvement in 34.8% vs. 43.7%, auricular involvement in 73.9% vs. 62.7%, external ear involvement in 60.9% vs. 45.8%, tracheobronchial involvement in 65.2% vs. 83.8%, laryngeal involvement in 41.3% vs. 50.0%, arthralgia involvement in 50.0% vs. 47.9%, chest wall involvement in 41.3% vs. 61.3%, renal involvement in 4.3% vs. 2.1%, central nervous system (CNS) involvement in 28.3% vs. 9.9%, and peripheral nervous system involvement in 4.3% vs. 2.1%. The clinical features and laboratory findings of the patients are presented in **Table 1**.

**Table 1:** Baseline clinical features between patients with and without cardiac involvement.

	Patients with cardiac involvement (n=45) percent/mean (range)	Patients without cardiac involvement (n=142) percent/mean (range)
Age	51.52(10-74)	43.33(12-72)
Male	60.9	54.2
Obesity	10.8	8.6
Diabetes	6.5	2.8
Hypercholesterolaemia	6.5	7.0
Hypertension	15.2	6.3
Smoke	43.5	38
Alcohol	34.8	23.9
Disease duration	26.17(0.17-20)	21.08(0.17-30)
Ocular	52.2	35.9
Nasal	52.2	57.7
Saddle Nose	34.8	43.7
Auricular	73.9	62.7
External ear	60.9	45.8
Tracheobronchial	65.2	83.8
Laryngeal	41.3	50.0
Renal	4.3	2.1
Arthralgia	50.0	47.9
Peripheral arthropathies	39.1	38.7
Chest wall	41.3	61.3
PNS	4.3	2.1
CNS	28.3	9.9
Skin	28.3	18.3
WBC	9.89(2.93-26.2)	9.37(2.85-22.07)
NEUT	7.38(1.32-20.63)	6.83(2.15-17.36)
PLT	310.04(48-893)	330.94(101-680)
Hb	121.87(65-159)	126.54(65-163)
ALT	26.33(4-133)	26.45(5-221)
Alb	36.55(13.5-53)	37.98(24-48)
LDH	200.47(100-530)	199.22(91-530)
Cr	75.88(40-499)	64.51(37-99)
CK	82.75(8-833)	43.62(9-111)
Fer	486.03(14-1500)	277(8-1174)
CRP	50.08(0.09-235.11)	54.69(0.13-312.24)
ESR	52.44(2-140)	49.32(1-140)
CAR	2.76(0-16.74)	2.76(0.05-10.72)
PLR	219.35(71.64-834.58)	206.26(46.67-888.89)
NLR	5.64(1.11-24)	4.38(0.88-18.66)

CNS, central nervous system; PNS, peripheral nervous system; WBC, white blood cell; NEUT, neutrophil; Hb, haemoglobin; PLT, platelet count; ALB, Albumin; ALT, alanine transaminase; LDH, lactate dehydrogenase; Cr, Creatinine; ESR, erythrocyte sedimentation rate; CRP, C-reactive protein; Fer, Ferritin; NLR, Neutrophil to lymphocyte ratio; PLR, platelet to lymphocyte ratio; CAR, C-reactive protein to albumin ratio.

The primary associated cardiac involvement was valvular insufficiency (n = 28, 62.2%), including aortic valve insufficiency (n = 17, 37.8%), mitral valve insufficiency (n = 9, 20.0%), and tricuspid valve insufficiency (n = 9, 20.0%). While three patients had aortic valve insufficiency with mitral valve insufficiency, two had mitral valve insufficiency combined with tricuspid valve insufficiency, and one had aortic valve insufficiency combined with mitral and tricuspid valve insufficiency.

Notably, one patient suffered from severe aortic insufficiency, combined with aneurysmal dilatation of the aortic sinus two months after the onset of the disease, and underwent Bentall’s surgery to achieve improvement. In addition to valvular involvement, some patients developed pericarditis (n = 11, 24.4%), ascending aortic aneurysm (n = 1, 2.2%), aortic root dilatation or thickening (n = 12, 26.7%), pulmonary hypertension (n = 3, 6.7%), and right bundle branch block (n = 1, 2.2%).

### 3.2 Univariate and multivariate analyses to define factors associated with cardiac involvement

Based on the clinical data of 187 patients, 36 clinical factors were included in the univariate logistic regression, of which 16 continuous variables were transformed into categorical variables as a supplementary analysis for inclusion in the univariate logistic regression.

**Figure 1:**
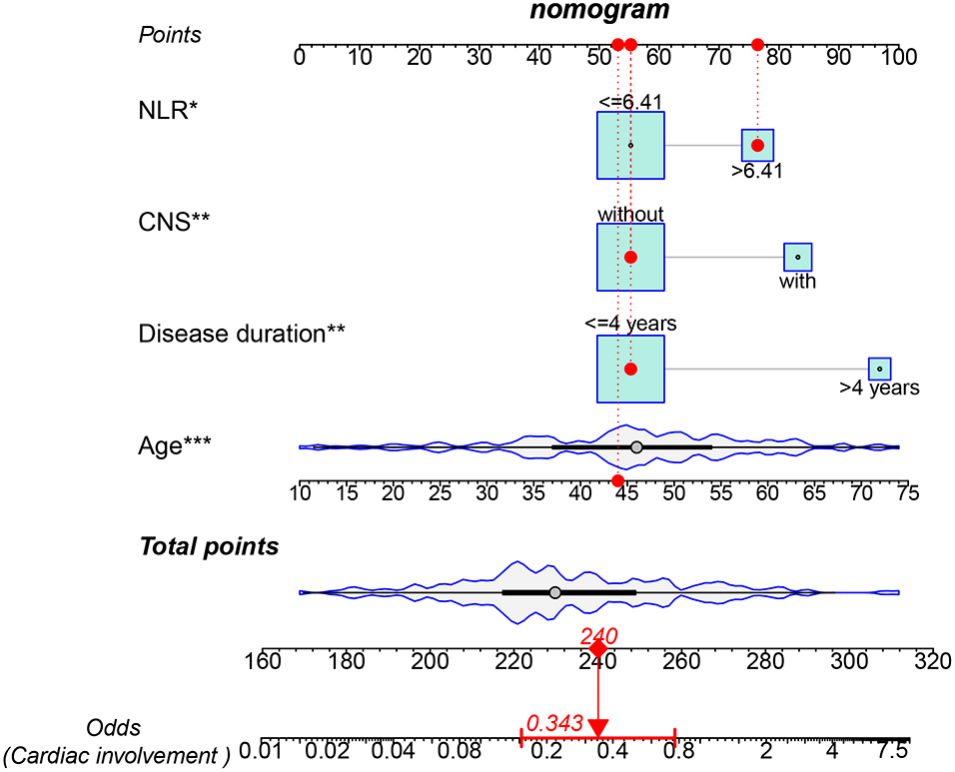
Nomogram for predicting cardiac involvement probability in patients with RP. Instructions on using nomogram: an individual patient’s value is located on each variable on the corresponding axis, and a line is drawn upward to determine the number of points received for each variable value. The sum of these numbers is located on the total points axis, and a line is drawn from the total points axis to the lower line of the nomogram for determining the cardiac involvement probabilities *p < 0.05, **p < 0.01, ***p < 0.001.

The results showed that four factors, including age (OR: 1.063, 95%CI: 1.029–1.099, p < 0.001), CNS involvement (OR: 3.602, 95%CI: 1.545–8.396, p = 0.003), NLR > 6.41 (OR: 2.669, 95%CI: 1.216–5.858, p = 0.014), and disease duration > 4 years (OR: 2.758, 95%CI: 1.017–7.477, p = 0.046), were positively correlated with cardiac involvement. Conversely, tracheobronchial involvement (OR: 0.362, 95%CI: 0.171–0.77, p = 0.008) and chest wall involvement (OR: 0.445, 95%CI: 0.226–0.876, p = 0.019) were negatively correlated with cardiac involvement (**Table 2**). Subsequently, multivariate regression analysis confirmed that age (OR: 1.071, 95%CI: 10.35–1.109, p < 0.001), CNS involvement (OR: 3.412, 95%CI: 1.354–8.601, p = 0.009), NLR > 6.41 (OR: 2.539, 95%CI: 1.076–5.993, p = 0.033), and disease duration > 4 years (OR: 6.220, 95%CI: 1.876–20.629, p = 0.003) were independent adverse factors for cardiac involvement. In addition, we constructed a nomogram consisting of four factors that provided quantitative predictive classifiers to identify the occurrence of cardiac involvement (**Figure 1**).

Table 2AUnivariate analyses to identify factors associated with RP patients with cardiac involvement in categorical variables.

**Table:** 

	Univariate analysis	
	OR (95% CI)	P
Age	1.063 (1.029-1.099)	<0.001
Gender	0.762 (0.387-1.500)	0.431
Obesity	1.285 (0.378-4.368)	0.688
Diabetes	2.407 (0.518-11.178)	0.262
Hypercholesterolaemia	0.921 (0.242-3.501)	0.904
Hypertension	2.652 (0.928-7.581)	0.069
Smoke	1.254 (0.639-2.460)	0.511
Alcohol	1.694 (0.826-3.476)	0.151
Ocular	1.947 (0.994-3.813)	0.052
Nasal	0.798 (0.409-1.556)	0.508
Saddle nose	0.688 (0.345-1.374)	0.289
Auricular	1.687 (0.804-3.539)	0.166
External ear	1.843 (0.935-3.630)	0.077
Tracheobronchial	0.362 (0.171-0.770)	0.008
Laryngeal	0.704 (0.359-1.379)	0.306
Arthralgia	1.088 (0.560-2.116)	0.803
Peripheral arthritis	1.017 (0.514-2.010)	0.962
Chest wall	0.445 (0.226-0.876)	0.019
CNS	3.602 (1.545-8.396)	0.003
PNS	2.106 (0.341-13.012)	0.423
Renal	2.106 (0.341-13.012)	0.423

CNS, central nervous system; PNS, peripheral nervous system; P < 0.05 was considered to indicate a statistically significant difference.

**Table 2B:** Univariate analyses to identify factors associated with RP patients with cardiac involvement in continuous variables.

Univariate analysis (continuous)			Univariate analysis (categorical)		
	OR (95% CI)	P		OR (95% CI)	P
WBC	1.038 (0.951-1.132)	0.405	WBC [(<3.5 and >9.5) vs (3.5-9.5)]	1.115 (0.572-2.177)	0.749
NEUT	1.047 (0.953-1.149)	0.341	NEUT [(<2 and >7.5) vs (2-7.5)]	0.987 (0.500-1.951)	0.971
Hb	0.989 (0.973-1.005)	0.172	Hb (male: <120; female <110 vs male: >=120; female >=110)	1.078 (0.522-2.226)	0.84
PLT	0.999 (0.996-1.001)	0.364	PLT [(<100 and >350) vs (100-350)]	0.896 (0.447-1.795)	0.756
Alb	0.957 (0.903-1.014)	0.137	Alb (<35 vs >=35)	1.264 (0.586-2.727)	0.551
ALT	1.000 (0.986-1.014)	0.975	ALT (>40 vs <=40)	0.818 (0.310-2.161)	0.685
LDH	1.000 (0.996-1.005)	0.922	LDH (>270 vs <=270)	1.087 (0.397-2.972)	0.872
Cr	1.011 (0.993-1.030)	0.231	Cr (>84 vs <=84)	1.944 (0.716-5.277)	0.192
ESR	1.002 (0.994-1.011)	0.641	ESR (>20 vs <=20)	0.713 (0.353-1.440)	0.346
CRP	0.999 (0.994-1.004)	0.692	CRP (>8 vs <=8)	0.714 (0.349-1.461)	0.357
Fer	1.002 (1.000-1.003)	0.087	Fer (>200 vs <=200)	0.947 (0.258-3.484)	0.935
NLR	1.077 (0.996-1.164)	0.063	NLR (>6.41 vs <=6.41)	2.669 (1.216-5.858)	0.014
PLR	1.001 (0.998-1.003)	0.571	PLR (>178.33 vs <=178.33)	1.540 (0.788-3.009)	0.207
CAR	1.014 (0.884-1.164)	0.839	CAR (>0.14 vs <=0.14)	0.595 (0.288-1.227)	0.159
Disease duration	1.031 (0.947-1.122)	0.487	Disease duration (>4 vs <=4)	2.758 (1.017-7.477)	0.046

WBC, white blood cell; NEUT, neutrophil; Hb, haemoglobin; PLT, platelet count; ALB, Albumin; ALT, alanine transaminase; LDH, lactate dehydrogenase; Cr, Creatinine; ESR, erythrocyte sedimentation rate; CRP, C-reactive protein; Fer: Ferritin; NLR, Neutrophil to lymphocyte ratio; PLR, platelet to lymphocyte ratio; CAR, C reactive protein to albumin ratio;P < 0.05 was considered to indicate a statistically significant difference.

### 3.3 Cardiac involvement may not be associated with RP prognosis

We compared the relationship between cardiac involvement and RP prognosis in several aspects. The results showed that cardiac involvement did not increase the rate of recurrence (OR: 0.584, 95% CI: 0.246–1.388 p = 0.224), mortality (OR: 2.720, 95% CI: 0.693–10.68 p = 0.152), or poor prognosis (OR: 0.837, 95% CI: 0.386–1.816 p = 0.653). We subsequently compared the differences in remission between the two groups, and found no significant differences in 1-year remission (OR: 1.596, 95%CI: 0.623–4.086, p = 0.33), 2-year remission (OR: 1.316, 95%CI: 0.467–3.710, p = 0.604), or 3-year remission (OR: 1.103, 95%CI:0.378–3.219, p = 0.167). Furthermore, cardiac involvement did not increase the rate of secondary hospitalization events (OR: 0.412, 95% CI: 0.117–1.450, p = 0.167).

### 3.4 Identification of clinical patterns of RP

**Figure 2:**
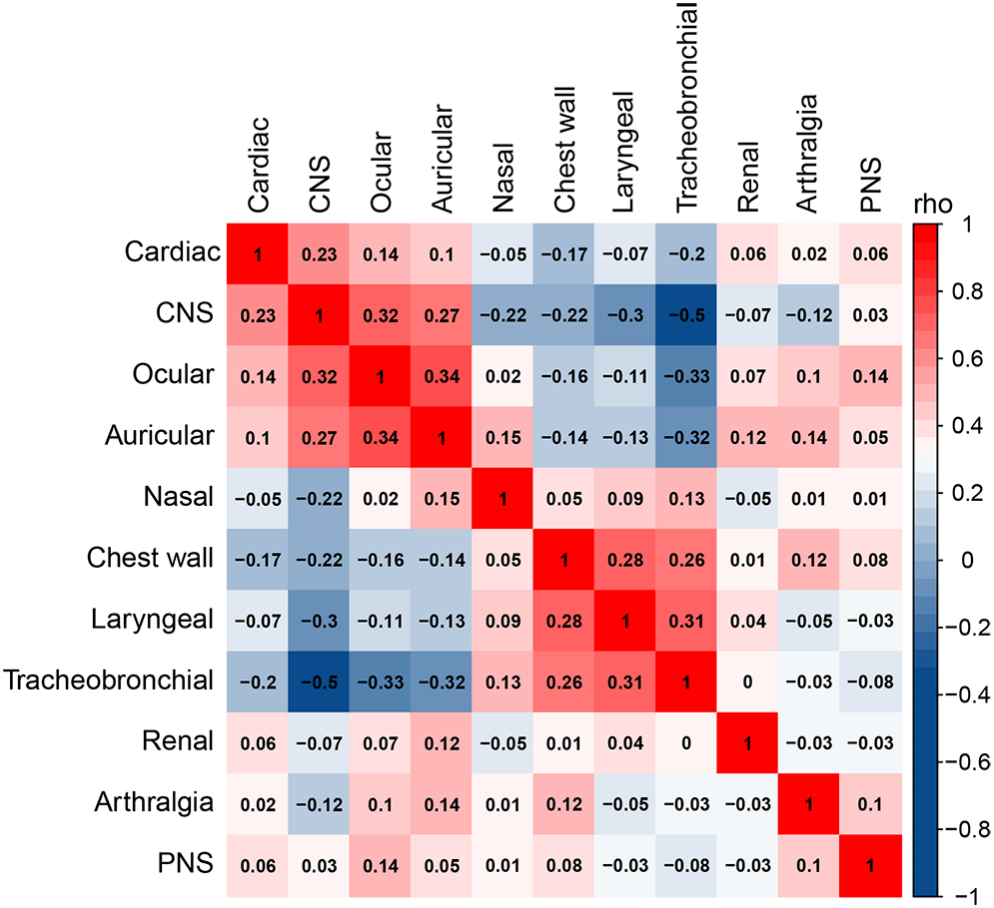
Correlation analysis of different organ involvement. Spearman’s correlation coefficient is used to measure the correlation between two variables, where a value closer to 1 indicates a stronger positive correlation, a value closer to -1 indicates a stronger negative correlation, and a value closer to 0 indicates a weak correlation.

RP is characterized by a remarkable heterogeneity, and our results revealed a close association between cardiac, CNS, tracheobronchial, and chest wall involvement. Accordingly, we analyzed the correlations between different organ involvement and constructed a correlation matrix, which was clustered based on the “hclust” algorithm (**Figure 2**), identifying five well-defined clinical patterns, including the heart-brain pattern (involving the heart, CNS, ear, and eye), airway pattern (involving all airway involvement), kidney pattern, joint pattern, and peripheral nerve pattern. Notably, the heart-brain and airway patterns were significantly mutually exclusive, with a negative correlation.

## 4 Discussion

In the present study, we divided patients with RP into two groups based on the presence or absence of cardiac involvement. Subsequent comparison of the clinical differences between the two groups revealed several results worth discussing.

In our study, cardiac involvement in patients with RP was mostly subclinical, and patients were assessed for cardiac involvement by performing transthoracic echocardiography and electrocardiography. In our study, the incidence of cardiac involvement was 24.1%, while that of valvular involvement was 16.0%. These rates are similar to those found in previous studies (9, 10). Cardiac involvement is not unusual for RP; although the majority of patients have subclinical changes, given that cardiac involvement can lead to fatal cardiovascular complications (e.g., complete heart block, aortic valve rupture, and acute aortic valve insufficiency), regular ECG and echocardiography should be performed to monitor cardiac involvement (9).

To determine the clinical differences between the two more accurately, we selected the common clinical features and risk factors of cardiovascular disease for inclusion in univariate and multifactorial logistic regression, finding that cardiac involvement was positively associated with age, disease duration, CNS involvement, and NLR, and negatively associated with tracheobronchial involvement and chest wall involvement. Based on the above outcomes, a line graph was created to digitally assess the risk of cardiac involvement.

A retrospective study in Japan showed that the mean age of patients with and without heart involvement was 72 and 65 years, respectively (11). In this study, the mean age of onset was 51 years in the group with cardiac involvement, compared to 43 years in the group without cardiac involvement; the difference was significant (p < 0.001). These findings suggest that the risk of cardiac involvement increases with age. Previous studies have shown a higher incidence of cardiovascular events in men than in women (12), while Shimizu et al. found that men with RP are at greater risk for heart involvement (11). However, in this study, no significant difference was found in the incidence of cardiac involvement in RP patients in terms of sex. We further analyzed other common risk factors for cardiovascular disease, such as hypertension, diabetes, hypercholesterolemia, and obesity but found that none of them were statistically different.

Our data indicate that a longer disease duration is associated with a greater risk of cardiac involvement. As a flare-up and interventional disease, the amount and extent of tissue damage increases over time (13). Similarly, a case report previously reported a patient with RP who developed an acute aortic lesion despite long-term remission (14). In addition, one study showed that diagnosis of an ear biopsy can be useful even in the absence of ear symptoms during the non-inflammatory interval (15). This suggests that despite the absence of both subjective symptoms and evidence of systemic inflammatory activity in patients, inflammatory activity may be observed at the organ level. Assessments of RP disease activity often focus on clinical symptoms, systemic markers of inflammation (e.g., CRP and ESR), and radiological investigations. We recommend that careful cardiac investigations, such as echocardiography and electrocardiography, should be performed regularly in patients with RP, both in flare conditions and long-term remission states.

The NLR describes the ratio of two opposing but supplementary immune pathways. This reflects the key role of non-specific immune responses, as with specific immune responses in inflammation (16). NLR, a novel inflammatory marker, has been proven to be associated with the activity of diverse immune diseases, such as SLE, primary Sjögren’s syndrome, Behçet’s disease, ankylosing spondylitis, polymyalgia rheumatic (17–21). A previous study by our team also revealed an association between NLR and disease activity in RP (22). Inflammation is often implicated in the pathogenesis of cardiac pathology, and various inflammatory markers have been found to be associated with cardiac conditions. NLR also plays a prognostic role in the stratification and prognosis of various cardiovascular diseases (23, 24). NLR is associated with severe valve stenosis and the severity of rheumatic mitral valve stenosis, as demonstrated in previous studies (25–27). Additionally, NLR is considered to be associated with poor prognosis in arrhythmias and heart failure, including relapses and mortality (28, 29). We suspect that the NLR is a reasonable predictor of cardiac involvement in RP because of its role in RP and cardiovascular disease.

In our study, we found that patients with cardiac involvement were more likely to have CNS involvement. Cardiac involvement was negatively associated with tracheobronchial and chest wall involvement in univariate logistic regression. Based on the multiplicity of clinical manifestations and the close correlation between different tissue involvements in RP patients, the exploration of diverse phenotypes of RP patients has been a hot research topic in recent years. Dion et al. first classified 142 RP patients into hematologic, respiratory, and mild forms using cluster analysis, finding that cardiovascular involvement occurred primarily in the hematologic form, with less cardiovascular involvement in the respiratory form (4). Subsequently, Shimizu et al. advocated dividing patients into a respiratory involvement group, an ear involvement group, and an overlapping group of both, and few patients with cardiac involvement were found in the subgroup of patients affected by respiratory involvement (30). Overall, the results of prior studies support the findings of the present study. We propose that cardiac and CNS involvement may indicate RP disease severity and that clinicians should therefore focus on the heterogeneity of RP patients, who should be examined in greater detail during clinical treatment.

We observed an inter-relationship between cardiac, central nervous system, tracheobronchial and chest wall involvement in RP patients. Specifically, the nasal, chest wall, laryngeal, and tracheobronchial systems had similar patterns of involvement, as did the cardiac, CNS, ocular, and auricular systems. This corresponds well with the studies by Ferrada and Shimizu et al. (30, 31). Further, these results suggest that identifying the heterogeneity of RP patients at an early stage could help to promote timely diagnosis, assessment, and treatment, thereby reducing target organ damage.

In our study, we found no indications to suggest an adverse prognostic effect of cardiac involvement, which may be related to differences in the definition of poor prognosis, and may also be related to sample differences. In conclusion, we believe that the prognostic role of cardiac involvement needs to be further discussed.

The prognosis of RP is of significant concern to clinicians and patients. Although Dion et al. suggested that cardiac involvement was a risk factor for mortality (4), studies in both China and Japan failed to find any significant association between cardiac involvement, death, and relapse (10, 32). In our cohort, we focused on the association of cardiac involvement with remission, flare-ups, and death, none of which showed significant associations, suggesting that cardiac involvement was not associated with prognosis. Further prospective cohort studies are required to examine the correlation between cardiac involvement and prognosis.

This study has limitations. First, the single-center, retrospective design may have led to potential bias, caused by incomplete information in some medical records and insufficient follow-up time. As such, large retrospective or prospective studies need to be carried out to validate these results. Secondly, our study only included Chinese patients and no other races. Therefore, whether the current results can be generalized to other populations requires further study.

## 5 Conclusions

Overall, we found that four clinical factors, namely, age, CNS involvement, NLR > 6.41, and disease duration > 4 years were risk factors, while tracheobronchial involvement and chest wall involvement were adverse factors for cardiac involvement. Regular echocardiography and electrocardiography are essential for patients with RP due to their significant cardiac impact.

## Data availability statement

The raw data supporting the conclusions of this article will be made available by the authors, without undue reservation.

## Ethics statement

The studies involving humans were approved by The Ethics Committee of Peking Union Medical College Hospital. The studies were conducted in accordance with the local legislation and institutional requirements. Written informed consent for participation in this study was provided by the participants’ legal guardians/next of kin. Written informed consent was obtained from the individual(s), and minor(s)’ legal guardian/next of kin, for the publication of any potentially identifiable images or data included in this article.

## Author contributions

RY, YPH, and YH contributed to the conception and design of the study. MZ, DX, QW and ML collected clinical data. RY, WZ and FZ performed the statistical analysis. RY wrote the first draft of the manuscript. All authors contributed to the article and approved the submitted version.
